# *Be good, communicate, and collaborate*: a qualitative analysis of stakeholder perspectives on adding a chiropractor to the multidisciplinary rehabilitation team

**DOI:** 10.1186/s12998-018-0200-4

**Published:** 2018-06-22

**Authors:** Stacie A. Salsbury, Robert D. Vining, Donna Gosselin, Christine M. Goertz

**Affiliations:** 1 Palmer Center for Chiropractic Research, Palmer College of Chiropractic, Davenport, IA USA; 2 Independent Consultant, Milford, NH USA; 3 Spine IQ – The Spine Institute for Quality, Davenport, IA USA

**Keywords:** Patient-centered care, Chiropractic, Rehabilitation, Interprofessional relations, Professional competence, Musculoskeletal pain

## Abstract

**Background:**

While chiropractors are integrating into multidisciplinary settings with increasing frequency, the perceptions of medical providers and patients toward adding chiropractors to existing healthcare teams is not well-understood. This study explored the qualities preferred in a chiropractor by key stakeholders in a neurorehabilitation setting.

**Methods:**

This qualitative analysis was part of a multi-phase, organizational case study designed to evaluate the planned integration of a chiropractor into a multidisciplinary rehabilitation team. The setting was a 62-bed rehabilitation specialty hospital located in the northeastern United States. Participants included patients, families, community members, and professional staff of the administrative, medical, nursing, and therapy departments. Data collection consisted of audiotaped, individual interviews and profession-specific focus groups guided by a semi-structured interview schedule. Transcripts were imported into a qualitative data analysis program for data analysis. An iterative coding process using thematic content analysis categorized key themes and domains.

**Results:**

Sixty participants were interviewed in June 2015, including 48 staff members, 6 patients, 4 family members, and 2 community members. Our analysis generated a conceptual model of The Preferred Chiropractor for Multidisciplinary Rehabilitation Settings composed of 5 domains and 13 themes. The central domain, Patient-Centeredness, or the provision of healthcare that is respectful, responsive, and inclusive of the patient’s values, preferences, and needs, was mentioned in all interviews and linked to all other themes. The Professional Qualities domain highlighted clinical acumen, efficacious treatment, and being a safe practitioner. Interpersonal Qualities encouraged chiropractors to offer patients their comforting patience, familiar connections, and emotional intelligence. Interprofessional Qualities emphasized teamwork, resourcefulness, and openness to feedback as characteristics to enhance the chiropractor’s ability to work within an interdisciplinary setting. Organizational Qualities, including personality fit, institutional compliance, and mission alignment were important attributes for working in a specific healthcare organization.

**Conclusions:**

Our findings provide an expanded view of the qualities that chiropractors might bring to multidisciplinary healthcare settings. Rather than labeling stakeholder perceptions as good, bad or indifferent as in previous studies, these results highlight specific attributes chiropractors might cultivate to enhance the patient outcomes and the experience of healthcare, influence clinical decision-making and interprofessional teamwork, and impact healthcare organizations.

**Electronic supplementary material:**

The online version of this article (10.1186/s12998-018-0200-4) contains supplementary material, which is available to authorized users.

## Background

Chiropractors are integrating into multidisciplinary settings with increasing frequency, but relatively little is known about the perceptions that medical providers and patients hold about the process of making this new addition to the healthcare team [1–4]. Perceptions about the chiropractic profession may differ considerably by stakeholder group [5–24]. Lay people often report being receptive to seeing a chiropractor as a patient [5, 6]. And yet, lay opinions about chiropractic are characterized by skepticism, confusion, and distrust on one extreme to enthusiastic affirmations about these providers on the other [5, 6, 25]. Chiropractic patients themselves often report positive evaluations of the care received from chiropractors, noting satisfaction with the clinical information offered, concern shown toward patients, and these providers’ confidence in treating back pain [8, 10, 26]. Chiropractic patient perceptions of the treatment abilities of chiropractors are strongest for musculoskeletal conditions, including back pain, muscle and joint pain, and headaches, with varying levels of support for the effectiveness of chiropractic treatment for other health conditions [11].

In contrast, the literature on interactions between chiropractors and other healthcare professionals often tells a story of fragmentation, disconnection, boundary skirmishes, and a general failure to communicate [12–17]. Primary care providers and medical specialists have recognized the competence of *some* chiropractors to treat *some* musculoskeletal problems in *some* patients, particularly those with low back pain [18, 19]. Medical and osteopathic physicians, physiotherapists, manual therapists, obstetricians and midwives, and other healthcare professionals often report minimal knowledge of the chiropractic profession or its treatments [18–20, 24, 27]. Further, some medical providers express concerns about the safety of spinal manipulation and voice skepticism over the efficacy of the therapeutic approaches used by chiropractors [18–20, 22, 27]. Healthcare providers and students often report having had no firsthand encounters with a doctor of chiropractic, either personally as a chiropractic patient or professionally in a collegial relationship [18, 24, 27], which may lead to misperceptions about the treatments offered by chiropractors. Nonetheless, many types of physicians describe negative attitudes towards chiropractic as a profession, at times based upon an experience reported by an individual patient [14, 19, 22]. For example, orthopedic surgeons report concerns with the variability in quality and approach between chiropractors, questioned the ethics of some providers and the use of ‘fringe’ treatments in some clinics, and commented on the inadequacy of educational training and the sparse scientific basis of chiropractic treatments [22, 23].

A commonality across these previous studies is the focus on public, patient, and provider perceptions about the chiropractic profession in general or as an abstraction, rather than within a specific healthcare context. Little is known about the perceptions that engaged stakeholders, or persons actively involved in the work of a healthcare organization, might hold toward the addition of a chiropractor to that particular facility, such as a clinic, hospital, or long-term care setting. To address this gap, our team conducted a multi-phased research project that supported and evaluated the introduction of chiropractic services into a rehabilitation specialty hospital/skilled nursing facility in the United States [28, 29]. This multimodal project included a long-term, organizational case study [30] designed to: 1) describe the perceptions of key stakeholders toward adding chiropractic care to the services provided to patients, and 2) evaluate how these perceptions change over the course of the multi-year project. The purpose of this qualitative analysis was to explore stakeholder perceptions of the qualities preferred in a chiropractor from the perspectives of patients, families, and interdisciplinary team members affiliated with this rehabilitation setting.

## Methods

This qualitative analysis was part of a larger organizational case study [30] that used ethnographic methods [31, 32] to explore the process of integrating a chiropractor into an established multidisciplinary team working in a neurorehabilitation setting. The ethnographic methods included short-term, immersive site visits consisting of participant observation, interviews, and focus groups, as well as ongoing, weekly interactions via conference calls with on-site clinicians and research team members [30, 31]. Supplemental information were collected from publicly available, on-line resources, such as the institution’s website, local media reports, and the Centers for Medicare and Medicare Services database, Nursing Home Compare (https://www.medicare.gov/nursinghomecompare/search.html?). This blended approach allowed investigators to understand better the emergent and sociocultural nature of the integration of the chiropractor into the rehabilitation team from the perspectives of those directly involved in this process. The current analysis reports on the baseline perceptions held by rehabilitation stakeholders before the introduction of chiropractic care services into the facility only. Other aspects of the process of integrating the chiropractor into this rehabilitation setting are planned for publication or presented elsewhere [29, 33].

### Ethics

The Institutional Review Boards of Palmer College of Chiropractic (2015 V166, Approval date April 20, 2105) and Crotched Mountain Foundation (no approval number per institutional process) provided the ethics approvals for this study. All participants signed a written informed consent before the start of the interview.

### Study setting

The study setting was the Crotched Mountain Specialty Hospital (CMSH), a 62-bed skilled nursing facility located in Greenfield, New Hampshire, in the northeastern United States. CMSH specialized in the sub-acute rehabilitation of patients with complex neurological conditions, including traumatic brain injury, spinal cord injury, and cerebrovascular accident. Some patients also were admitted for management of long-term ventilator dependency. CMSH provided in-patient services for both adult and pediatric patients, although the focus of this project was on the integration of chiropractic care for adult patients only. Adult in-patients resided on one of three nursing units, where the care was tailored to the admitting diagnoses (e.g., brain injury unit). Patient-centered treatment was delivered by a multidisciplinary team composed of medical physicians (internist, pediatricians, psychiatrist, and physiatrist) and nurse practitioners, physical and occupational therapists (PT/OT) and assistants (PTA/OTA), speech therapists (ST), psychologists, registered nurses (RN), therapeutic recreation therapists and assistants (TR/TRA), assistive technology staff, and licensed nursing assistants (LNA). Patient care was supported further by non-clinical staff in the housekeeping, dietary, maintenance, and other departments. The facility was a non-profit corporation that participated in the Medicare and Medicaid programs for support of older and disabled persons, and people with low incomes, respectively.

### Participants and recruitment procedures

Our study sample included representatives of key stakeholder groups of CMSH. We recruited a purposive sample for interviews and focused groups that included patients, family and community members, administrative personnel, and members of the clinical team, including medical doctors, nursing staff, and therapy staff. Inclusion criteria for participants were English-speaking adults over the age of 18 years old who were stakeholders in the rehabilitation hospital and who were willing to consent to an audio-recorded interview or focus group session. While no minimum sample size for this study was determined a priori, our goal for participant recruitment was to invite all persons who likely would interact with the chiropractor in their working relationship, or who could offer an informed opinion on how the chiropractor should best be integrated into the facility, to participate. In addition, all persons from specific job classifications, such as all members of the therapy department and all medical providers, were recruited to participate given the likelihood of close interaction with the chiropractor.

The presence of a health condition of such severity as to prevent the individual from communicating verbally during the interview process (e.g., aphasia, profound hearing loss, coma, etc.) was the major exclusion to enrollment. We have described the demographic and clinical characteristics of the CMSH patient population elsewhere [29]. Males (67%) with a mean age of 42.8 years with a history of brain injury (74%) comprised most participants. All participants in this qualitative study were their own legal representatives for healthcare decisions who signed informed consents to be interviewed. However, like many CMSH patients [29], some participants in this qualitative study had cognitive impairments and communication challenges, including difficulties with verbal expression and fatigue during extended verbal interactions. As such, the quotations from patients were generally shorter in length compared to those from other participants, and more limited in their word choices. Readers are asked to keep these aspects of the patient population in mind when reading their quotations.

Participants were recruited by CMSH co-investigators through personal invitations and by using brochures designed for patients/families or staff members. Nursing and therapy staff were offered light refreshments during their focus groups to enhance recruitment as the sessions were scheduled during typical work breaks. Departmental administrative staff ‘covered the floor’ to assure on-going patient care during interview sessions. These clinical staff were given a $25 gift card to compensate for lost work time as most had to extend their work hours on the day of participation. All other participants received no financial or non-monetary incentive to enroll in the project.

### Data collection procedures

Data collection consisted of individual interviews and focus groups conducted in June 2015, about 2 months before the hiring process began and 4 months before the chiropractor started orientation. We described the hiring and orientation process of the chiropractor elsewhere [29]. The chiropractor was interviewed during the first weeks of orientation (October 2015). Fieldnotes, publicly available data about the institution, and research team meeting minutes supplemented interview data. Most data collection sessions were conducted by the lead author (SAS), with several focus groups co-moderated and one staff interview completed by the co-principal investigator (RDV). The interview team had little previous contact with the majority of CMSH staff members prior to their interview, and no previous contact with the patients or families interviewed. No CMSH investigators were involved in the conduct of the interviews.

Participants received an informational brochure outlining the main details of the study, an informed consent document, and a verbal overview of the study purpose and procedures before the start of the interview. Individual interviews were held with patients, families, community members, administrative personnel, medical physicians, and a nurse practitioner. Role-specific focus groups were convened for members of the therapy department (PT, OT, PTA/OTA, ST, TR/TRA, psychologists, and adaptive technology engineers) and nursing staff (RN and LNA). Managers from therapy and nursing were interviewed in small groups by department, separately from the clinical staffs. All focus groups and most interviews were conducted in person at CMSH in conference rooms, offices, patient rooms, or unoccupied lounges at the facility. Two interviews, one conducted with an administrator and one with the chiropractor, were completed as telephone or videoconference interviews. All sessions were audiorecorded using digital recorders (Sony ICD-UX71, Tokyo, Japan; OlympusWS-801, Tokyo, Japan), with large group interviews recorded with 2 devices to assure proper reception.

A semi-structured interview manual guided the sessions, with interview topics varying somewhat by participant role. For example, patient and family interviews focused on the patient’s experience of their injury or illness and the rehabilitation process; issues with pain or functional impairments and treatments received for these conditions; and any previous involvement with chiropractic care. In contrast, interviews with staff members highlighted their direct experience of caring for rehabilitation patients and their personal and/or professional perceptions of chiropractic care. All stakeholders were asked their specific recommendations for initiating and sustaining a chiropractic program in this setting. Interview topics, while guided by the manual, were introduced to participants with a flexible sequence to follow the natural flow of conversation, especially in the focus group sessions. While stakeholder perspectives about chiropractic might be voiced at any time during the interview, and were coded as such when identified in the written transcripts, two questions garnered the most discussion among participants about the preferred qualities of the chiropractor who would soon join the well- established multidisciplinary team:

1. What does a chiropractor need to know about this setting to work well with the patients?
2. What does a chiropractor need to know about this setting to work well with the other staff?

### Data analysis

Audiorecordings were transcribed verbatim by a transcription service (Way With Words, New York, NY, USA), with transcripts reviewed for accuracy by the lead author (SAS) and imported into NVivo® (Version 9.2, QSR International Pty Ltd., Victoria, Australia) for data management and analysis. Data analysis was completed by a research team, including the lead author who is an experienced qualitative investigator and 3 chiropractors who were learning about qualitative methodology as fellowship trainees in a masters of clinical research program. The team conducted qualitative content analysis using a conventional approach in which codes are identified inductively from the data during analysis [34]. Team members read all transcripts in their entirety. Fellows independently coded each transcript on paper, then the entire team met to review the coding process and discuss discrepancies in the coding. Final coding decisions were entered as nodes in the data analysis software, with the team identifying labels, definitions, and descriptions for all new codes in the emerging codebook. Superordinate (parent nodes or domains) and subordinate (child nodes or themes) categories were developed as links between the various codes were identified. Repeated readings with constant comparison across the transcripts identified the similarities and differences in findings among participants and between stakeholder groups [34, 35]. Following completion of coding for all transcripts, a second round of coding was completed. During this round, the transcript texts were reviewed by smaller coding teams of 2–4 members, updated with the final codebook, with codes/themes combined, refined or edited as indicated.

## Results

**Fig. 1:**
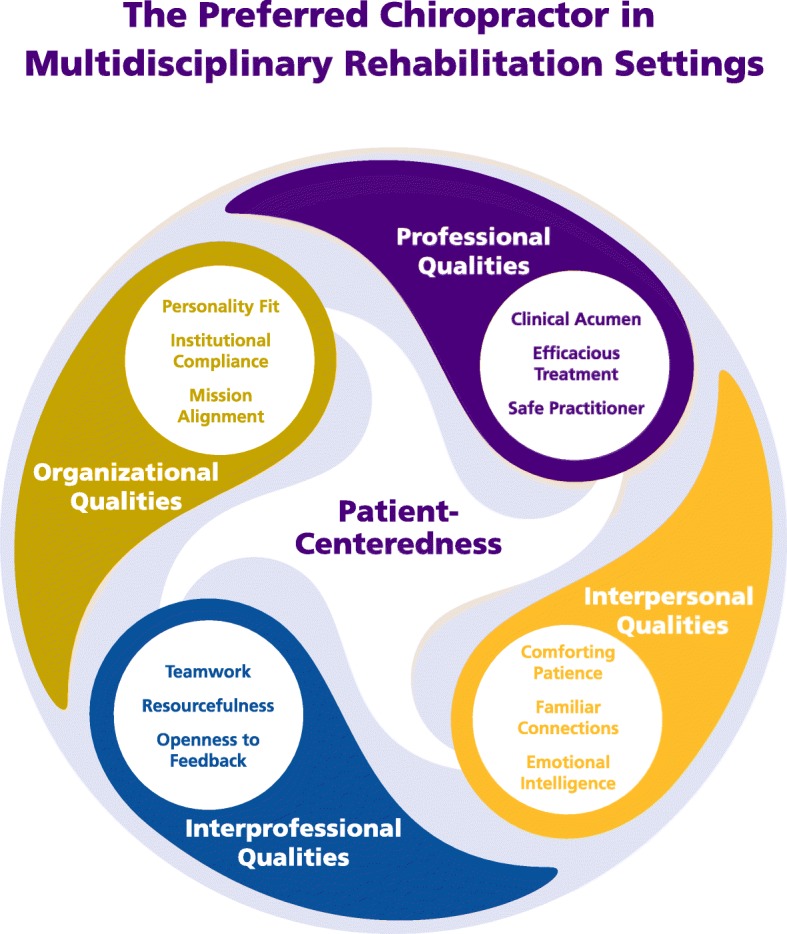
Conceptual model of the preferred chiropractor in multidisciplinary rehabilitation settings

Sixty participants were interviewed individually or in focus groups, including 48 staff members, 6 patients, 4 family members, and 2 community members. Rehabilitation hospital stakeholders identified many qualities they preferred in the chiropractor who soon would join their multidisciplinary setting. Our analysis generated a conceptual model of *The Preferred Chiropractor in Multidisciplinary Rehabilitation Settings* composed of 5 domains and 13 themes (Fig. 1). The central domain, *Patient-Centeredness*, was mentioned in all interview sessions, included the most references across interviews, and was linked most often with the other themes (Table 1). Additional qualities were categorized into 4 domains, with 3 themes undergirding each domain: *Professional* (clinical acumen, efficacious treatment, and safe practitioner)*, Interpersonal* (comforting patience, familiar connections, and emotional intelligence)*, Interprofessional* (teamwork, resourcefulness, and openness to feedback), and *Organizational* (personality fit, institutional compliance, and mission alignment). Illustrative quotes are included with the thematic presentation below, with additional quotes for each theme offered in the Additional file 1. For presentation, direct quotes are offered with an identifier for participant role and transcript number (e.g., P1 – Patient).

**Table 1:** Qualitative themes of preferred chiropractor in multidisciplinary rehabilitation settings by stakeholder group

Quality	Patients (6 Intvws)	Family or Community (5 Intvws)	Medical Staff (6 Intvws)	Therapy Staff (3 FGs)	Nursing Staff (4 FGs)	Administrative Staff (2 Intvws)	Total # Interviews (*n* = 26)	Total # References
Patient-Centeredness	6	5	6	3	4	2	26	140
Clinical Acumen	3	5	6	3	4	2	23	106
Teamwork	4	2	5	3	4	2	20	88
Efficacious Treatment	4	4	5	3	2	1	19	42
Comforting Patience	2	5	3	2	3	1	16	34
Personality Fit	1	1	5	3	3	2	15	47
Safe Practitioner	1	3	3	3	4	0	14	40
Familiar Connection	0	2	3	2	4	2	13	35
Institutional Compliance	2	1	3	2	2	2	12	38
Emotional Intelligence	0	1	3	2	3	1	10	47
Resourcefulness	0	0	3	3	2	1	9	46
Openness to Feedback	0	0	3	3	2	1	9	25
Mission Aligned	0	2	1	2	2	1	8	21

# Interviews = Total number of interviews or focus groups in which theme was mentioned

# References = Total number of times a theme was mentioned across all interviews

Intvws = Interviews

FGs = focus groups

### Patient-centeredness

*“You have to be willing to put your own agenda behind what needs to happen for that patient on that given day”* (M6 – Medical Staff).

*Patient-Centeredness*, the central domain, was defined as the quality of a chiropractor (and, importantly, all staff members) that demonstrates a provision of care that is respectful and responsive to the patient, and which is inclusive of the person’s values, preferences, and needs (Fig. 1). Each patient who was interviewed identified at least one instance of patient-centeredness that they had experienced with current staff members. An exemplar of this attitude came from a patient’s description of his work with a physical therapist:

*“Patient oriented. He makes you part of the program. You know exactly what’s going on and why he is doing what he’s doing”* (P1 – Patient).

Many patients further specified how they expected the chiropractor to demonstrate this same quality in their interactions. For instance, since no two patients were alike, patients and staff thought the chiropractor should have personal knowledge of each patient as well as information about the history of their injury and his or her current medical conditions. Such personal knowledge should then be integrated into the evolving care of the individual patient.

*“Every patient here has their own story, so what is good for one person may not be good for another person”* (P5 - Patient).

*“There is variability not just patient to patient but within the same patient as they may not be consistent”* (T7 – Therapy Staff).

Some participants expressed that in the neurorehabilitation context, families, too, should be included in decisions about the delivery of patient-centered care:

*“When you’re dealing with folks with brain injuries, it’s really important to not only ask the patient what might be needed or how the approach might be, but I think it’s really good to check in with the family members, too, to see if that would be a good thing… Sometimes things have got lost in the translation”* (FC – Family Member).

Patient preferences about their healthcare delivery were important considerations. For example, different patients might have previous preferences or expectations about chiropractic care, while others might have none. Patients who had received chiropractic care in the past might need new information about how their injury could change the delivery of chiropractic services. Or, simply, patients might prefer to schedule their chiropractic visits at varying times of the day.

*“They’d* [the chiropractor] *have to know their* [patient] *limitations and their desire to maybe be limbered up a little bit with exercise and different movements”* (P3 – Patient).

*“Different times for different people, you know. Some people are morning people, some people are later in the day people”* (NMU1 – Nursing Staff).

Staff members noted that it will take the chiropractor time to learn all of these details about each patient, but cautioned against rushing this process. The impact of the patient-centered approach could make a big difference in the person’s recovery:

*“One thing we forget a lot of times* [when] *doing their* [the patient’s] *care, it is not our pace. It is*** their*** pace. People coming from the outside in, it is one of the hardest things to learn. It is not about us, it is about them. That person can do it in two minutes. That one takes ten. You need to give them that, because you’re knocking them down a peg when you don’t. Everyone is different, there’s no two cases alike here, no two alike”* (NMU1 – Nursing Staff).

From this central characteristic of patient-centeredness, stakeholders in the rehabilitation process noted several other preferred qualities in a chiropractor. The first domain discussed are those qualities an individual would bring to the hospital as a healthcare professional with training in chiropractic.

### Professional qualities

The domain, *Professional Qualities*, was defined as the characteristics of the chiropractor that demonstrated his or her clinical knowledge, competence, and proficiency in the specialized field of chiropractic. Three professional qualities preferred by rehabilitation stakeholders were clinical acumen, efficacious treatment, and being a safe practitioner.

**Clinical acumen** was the ability to make good judgments and decisions about chiropractic care and the patient’s health concerns. That is, patients and providers wanted the chiropractor to be an expert and experienced healthcare professional with a broad knowledge base about chiropractic care and a deep understanding of both musculoskeletal conditions **and** neurological conditions such as traumatic brain, stroke, and spinal cord injury. The chiropractor should also possess proficiency in delivering a wide array of specialty-related treatments. For patients, this clinical acumen might be simply stated as *“to take care of the pain issues”* (P4 – Patient) or more complexly articulated as:

*“To understand what my specific therapeutic requirements are over and above that I am able to currently receive here”* (P1 – Patient).

As this patient noted, the chiropractor would need to understand how chiropractic care might be best applied to the individual’s case and how chiropractic therapies could augment or interact with the medical and therapy treatments the patient was prescribed. Staff members also stressed the importance of understanding the clinical presentation of various brain injuries and how to work with patients who have had such major changes to their neuromusculoskeletal systems. In these cases, clinical acumen was required to evaluate, to communicate, to move, to treat, and even to select patients who might benefit from chiropractic care in this setting:

*“A lot of patients with head injuries have a difficult time controlling their behaviors and their moods and emotions and I think that would be a challenge potentially…with the hands-on care that chiropractic care involves…there are just so many different presentations here, you’d have to really pick the people that are suited to get that modality. I don’t think everybody would be a candidate”* (M2 – Medical Staff).

After using clinical acumen to identify the appropriate candidates for chiropractic care, rehabilitation stakeholders were concerned that the chiropractor should use only those therapeutic modalities that were likely to improve patients’ health status. Stakeholders were unsure as to what forms of chiropractic care might be most beneficial to rehabilitation patients. However, this **efficacious treatment** was described as delivering treatment modalities that would provide a discernable, therapeutic impact on patient outcomes, including pain and disability. As one patient, a middle-aged man who had suffered a traumatic brain injury, stated of his hope for patient improvements with chiropractic care:

*“They* [patients] *get better…* [he should] *do that thing he* [chiropractor] *does. Hopefully he can put them back in place and make them work better”* (P4 – Patient).

A member of the administrative staff described the expected results from chiropractic care as:

*“The first starting point is that* [chiropractic care] *will have immediate value, or direct value, in comfort, capacity, and functional opportunity for individuals”* (A1 – Administrative Staff).

Of importance to the clinical staff at this facility was their ability to offer patients multiple treatment options, both as individual providers and collectively as a multidisciplinary team: “*lots of tools in your repertoire”* (T2 – Therapy Staff). Medical staff hoped that the addition of chiropractic care would offer patients effective, evidence-based options for the treatment of their pain beyond the medications usually prescribed:

*“It’ll be nice to treat a lot of the pain stuff or the musculoskeletal stuff with other modalities…People are excited about things that can help them”* (M2 - Medical Staff).

*“The successful integration of a chiropractor would be that they had something to offer to the patient that would help them on that* [recovery] *pathway”* (M5 – Medical Staff).

Many stakeholders were concerned that the chiropractor would be a **safe practitioner**, demonstrating a focus on patient safety through keeping patients from harm, preventing errors, and recognizing adverse events. Some participants expressed concerns with the safety of specific techniques: *“Would you use the snap, crackle? I don’t know about that one”* (C2 – Community Member). Other stakeholders worried about the delivery of chiropractic care to patients who have had spinal cord injuries:

*“The nerve-wracking part of it, because we have a lot of people that have spinal cord swelling. I feel like if, I don’t know, just one wrong movement could cause more damage instead of relief”* (NH3 – Nursing Staff).

One patient wondered if chiropractic care might exacerbate the current symptoms, potentially causing more harm than symptom relief to the patient:

*“So that would be the thing I’d be afraid of, is getting the chiropractor even to come close to some people without it hurting more”* (P2 – Patient).

Stakeholders clarified 3 qualities in the professional domain important for chiropractors to bring to the rehabilitation domain: clinical acumen within the fields of chiropractic and neurorehabilitation, treatments that would make a measurable impact on patient outcomes, and a focus on patient safety. From here, participants outlined the essential interpersonal characteristics a chiropractor might offer patients recovering from neurological insults and traumatic injuries.

### Interpersonal qualities

The *Interpersonal Qualities* domain were those characteristics of the chiropractor that will enhance his or her work with neurorehabilitation patients and their families. Themes in this domain included a comforting patience, familiar connections, and emotional intelligence.

A **comforting patience** was fundamental to the creation of a healing environment for the neurorehabilitation patient. Family members and patients alike noted the need for both a comfortable space for care interactions and a comforting attitude from care providers. Problematically, the therapy suites were known as places where patients pushed themselves in exercise, attempted to achieve therapeutic goals, and had limited opportunity for rest. The chiropractic visit was proposed as a time and a space within the rehabilitation setting where patients might experience relaxation and focus on self-care:

“*One thing that would definitely be beneficial is a very calm, quiet area where the patients could have their session with the chiropractor”* (FA – Family Member).

Participants recommended that the chiropractor take gentle and patient-centered approach to treatment delivery during the chiropractic visit and cautioned that patience may be necessary for some clients:

*“They’re* [the chiropractor] *going to have to be caring and loving and take time with the patients. I think they’ll do really well. As long as they* [the patients] *trust the person and it takes a little while”* (FD – Family Member).

Making **familiar connections**, or establishing rapport with the patients and families was an important interpersonal skill preferred in the chiropractor. Rapport could be established through a traditional doctor-patient relationship that focused on treatment of the health condition. However, in this longer-term, home-like setting, patients and staff often got to know one another on a personal level, through discussions about popular culture, sports teams, and hobbies. Current staff members were viewed as knowledgeable sources of such information about the patients, but it was the ongoing relationships built over time with the patient that were most often discussed. As was the case for other clinicians in this setting, stakeholders agreed that one chiropractor, integrated into the larger staff, would more likely establish such connections than could multiple chiropractors. As one mother stated:

*“I would like to see him* [my son] *have more…familiarity, so he gets comfortable with somebody”* (FD – Family Member).

**Emotional intelligence**, was described as the capacity to understand the emotions of others, be aware of one’s own emotions, and to manage emotions in interpersonal relationships [36]. Stakeholders noted that following a brain or spinal cord injury, patients were different people, with new patterns of emotional expression. Understanding these emotional changes was considered crucial to the chiropractor’s interactions with neurorehabilitation patients:

*“Have a thick skin, don’t take anything personally…the patient will have this anger, it’s being directed at you, but it’s not about you…it’s not them yelling at you for the day, it’s the fact that they can’t help what they do because of their brain injury…you have to remember that when you work with this population”* (NL5 – Nursing Leader).

Staff members also pointed out the brain injury may impact the patient’s outward behaviors, which could lead to some potentially intense verbal exchanges:

*“You don’t want to go in with a pre-conceived notion…’*[I] *know…what the story is’. Because sometimes they’re stuck and they’re very sensitive, very, very emotional. And they can be verbally aggressive”* (TL1 – Therapy Leader).

One mental health provider advised the chiropractor consider emotions and personality, in addition to cognitive abilities, when working with neurorehabilitation patients:

*“We think about people’s personalities and…what they’re likely to do and not do, and likely to agree with or not agree with, what kind of people they are…that’s valuable for anybody treating, working with somebody. It’s a human, intuitive, skill. If you’re in the healthcare field you probably have that skill on some level”* (M2 – Medical Staff).

Stakeholders proposed that a chiropractor working within neurorehabilitation setting would need interpersonal skills that included a demeanor that calmed patients, the ability to engage patients as individuals with their own interests and passions, and an understanding of the relationship between emotions and health. Beyond this, the new chiropractor would require interprofessional abilities to work with other providers on the healthcare team.

### Interprofessional qualities

*Interprofessional Qualities* were defined as the characteristics that will enhance the chiropractor’s ability to work with health professionals within an interdisciplinary setting. Three themes compromised this domain: teamwork, resourcefulness, and openness to feedback.

**Teamwork**, or the cooperative efforts of the clinical team toward their shared purpose and agreed upon goals in patient care, was a defining quality among CMSH providers. Each healthcare provider had specific contributions that he or she made toward the overall treatment plan for patients, but those individual offerings were downplayed against the backdrop of coming together and working as a team for the benefit of the patient:

*“We’re 95% team oriented here. We do everything as a team, even across shifts, so it’s all about team play here”* (NML3 – Nursing Staff).

Participants expected that the new chiropractor would join the clinical team as a full and active professional. However, chiropractic integration into the healthcare team meant different things to different stakeholders. For one patient, teamwork was an abstract ideal: *“Work well together, become part of a synergistic program”* (P1-Patient). For members of the therapy department, teamwork was a more concrete concern for everyday clinical practice. With a fairly small number of people comprising the clinical staff, team members often worked together delivering care to a single patient:

*“As chiropractors, are they comfortable co-treating, like with physical therapy or occupational therapy or speech* [therapy] *because there are lots of times when it does need two of us”* (T1 – Therapy Staff).

One participant noted that professional humility was inherent in successful teamwork in healthcare settings:

*“The whole effort here, when chiropractic goes on here, there should be a unity of integrated health services and…everybody gets credited with the results”* (C1 – Community Member).

A physician offered this straightforward expectation for a chiropractor joining the staff:

*“Be good and be able to communicate with a team and do their job well and collaborate. I think that would go a long way”* (M2 – Medical Staff).

**Resourcefulness**, or the ability to develop creative solutions to patient care challenges or technical problems, was required by the clinical staff as a ‘typical’ neurorehabilitation patient did not exist in this setting:

*“It’s a very diverse population. Nobody’s brain heals exactly the same from an injury so you’re constantly challenged by the presentation, so clinically, it’s very interesting”* (T1 – Therapy Staff).

To address the constantly changing patients, clinical staff demonstrated flexibility in thinking. Such resourcefulness in approach and persistence in addressing the unique needs of the individual were viewed by many staff members as valuable qualities in their colleagues, and an essential attribute in new team members:

*“A lot of different modalities are tried. People aren’t really geared here to give up. I think that’s the biggest thing, they’re not geared to give up, nobody ever gives up”* (NL1 – Nursing Leader).

*“Sometimes you go in with a very good game plan and you have to completely change so you have to be very flexible to work here… If you’re really going in with a plan, that’s not good, and you can’t mentally flex that plan in your head that’s not going to be a good fit”* (T4 – Therapy Staff).

Resourceful clinicians also show a willingness to change an approach that is not working, described by one participant with the tongue-in-cheek comment, *“he better be able to drive up the mountain road in snow”* (NL3 – Nursing Leader). A staff physician more seriously described this resourcefulness as:

*“I think maybe just an openness to seeing things differently and not just sticking to the protocol, within reason, and obviously, within the bounds of safety for the patient first”* (M1 – Medical Staff).

Such openness was not only desired in one’s intellectual viewpoint, but also in how providers interacted with other team members. **Openness to feedback**, or the acceptance and incorporation of new ideas or information into one’s practice, was the third theme of the interprofessional domain. As one department leader expressed, the variations in patients’ injuries and recoveries meant clinicians needed:

*“An openness to learn because you’re going to learn so much more…you’ve got to be open because you may have never done anything like that in the past…”* (TL2-Therapy Leader).

Other suggestions on this theme of openness for the new chiropractor were to *“ask questions and get feedback”* (T7-Therapy Staff) and:

*“Come to the same meetings that we all go to so that they can ask questions, learn our patients, know who does what and would be another addition to the team, as opposed to working out there by themselves”* (M5 – Medical Staff).

When working with other healthcare professionals in an in-patient rehabilitation setting, chiropractors were encouraged to engage in teamwork and collaboration, to demonstrate flexibility in thinking about clinical approaches to patient care, and to be open to the ideas of other members of the healthcare team. The final domain outlined stakeholders’ preferences for the chiropractor who was joining the boarder healthcare organization.

### Organizational qualities

The *Organizational Qualities* domain was comprised of the characteristics of the chiropractor, and all staff members, that were considered important attributes for working in this particular healthcare organization (but which may be transferrable to healthcare institutions more broadly). These themes included personality fit, institutional compliance, and mission alignment.

**Personality fit** included the personal traits that would allow an individual to adapt successfully to this specific organization. Administrators and clinical staff, rather than patients or families, identified personality fit as an important characteristic of the soon-to-be hired chiropractor. As a member of the medical staff noted, an individual who was a good listener and tolerant communicator would fit in well in this facility:

*“Personality is key. It’s going to need to be somebody who is going to interact well with the staff, will listen to the staff’s concerns, and will feel comfortable educating the staff – because none of us know anything about chiropractic – so that we can slowly develop a comfort level with it.”* (M5 – Medical Staff).

Specific personality-related qualities included an oft-mentioned sense of humor, an unselfish attitude, enthusiasm, accepting, easy going, persistent, caring, friendly, a high level of positivity and commitment, and a professional approach, with a personality that is just *“a little bit of quirky”* (NL1 – Nursing Leaders) being acceptable. Among the most preferred, and elusive, qualities of potential staff members at this facility was one of gratitude, or:

*“Finding joy in the smaller pieces, smaller rewards, is not something that everybody can do.”* (TL2 – Therapy Leaders).

Another theme identified under this domain was **institutional compliance**, or the ability to understand the rules of an organization, to work within this system, and to adhere to the administrative requirements of the position. Administrators and staff discussed issues with the credentialing process, insurance and reimbursement issues, and the use of electronic health records and other technologies. With the addition of this new type of healthcare provider onto the team, several medical staff were concerned with the annual review process, and how they would recognize whether the chiropractor was practicing according to their scope of practice:

*“To say, ‘Well, what does a good chiropractor do?’ I would not be able to look at a chiropractic chart and say, ‘this is basic standard of care, or this falls out…’ I wouldn’t know exactly”.* (M3 – Medical Staff).

To meet this concern, the facility established an evaluation system that included in-house medical staff who evaluated organizational behavior and compliance and relied on an external reviewer, a chiropractor and a member of the research team, to review clinical records, observe care, and provide feedback to administrators on whether standards of care were met. Another area of compliance noted by medical staff was provider attendance at scheduled meetings, such as the weekly interdisciplinary care team meeting:

*“It would be important for this person to come to the various groups that are already set up…interdisciplinary team is always Thursday at 8:30, because these are the systems that we’ve put in place to make sure that communication is open, all of those issues are discussed at those meetings”.* (M6 – Medical Staff).

Finally, many facility leaders tacitly advocated for **mission alignment** in the people who worked here. That is, they sought in a chiropractor a professional who, like themselves, might meet their personal goals through their work with the organization. Personal stories told of individual staff or community members who were *‘called to the mountain’* and described this quality of mission alignment:

*“From the president and down to someone that’s sweeping a floor, they just are wonderful, wonderful people….The good they do, it’s just remarkable. It totally is. They take people in that nobody else wants to work with or try to help. That’s what we like about it. It’s just doing such good work for so many people”.* (C2 – Community Member).

## Discussion

This qualitative study explored stakeholder perspectives on the professional and personal qualities of a chiropractor who prospectively would join the clinical staff of a rehabilitation specialty hospital in the U.S. Specifically, we sought to understand what healthcare professionals, patients, families, community members, and administrators thought the inclusion of a chiropractor would add to the institutional milieu and to the services offered to neurorehabilitation patients by an established healthcare team. Our results describe a preferred chiropractor for multidisciplinary rehabilitation settings as a patient-centered professional who possesses clinical acumen, practices in a safe manner, and is informed about efficacious and evidence-based treatments for this patient population. Such a chiropractor would draw upon an emotional intelligence to offer comfort to persons with painful conditions, patience to people with complicated recovery journeys, and a familiar connection to everyday life through a friendly rapport. Future colleagues anticipated working with a chiropractor who was a team player, resourceful in his or her approach to solving complex clinical problems, and responsive to formative feedback about their role in the facility and with patient management. The preferred chiropractor also would express a personality that fits with those in the larger organization, meet all procedural and legal requirements, and share in the mission and values of the institution.

These preferred qualities identified by rehabilitation stakeholders align with many proposed indicators of professional excellence outlined in a recent commentary on ‘the new chiropractic’ [37] and both old and new calls for chiropractors to assume a leading role in musculoskeletal health as spine care practitioners [38–40]. For example, the *clinical acumen* of chiropractic students might improve substantially through proposed hospital-based rotations that emphasize the evaluation and treatment of the musculoskeletal problems for persons with multiple comorbidities, such as neurological disorders [37]. Engagement in clinical experiences based in a wider array of healthcare settings than chiropractic clinics could increase chiropractors’ expertise working with patients who rely upon medical devices, adaptive technologies, and transfer equipment, as is common in rehabilitation settings, including the facility which served as this research site [28, 29]. Such hospital-based training also could increase chiropractors’ knowledge and application of the biopsychosocial model [29, 40], which might impact their proficiency within the *intrapersonal domain* as described by our participants. Multidisciplinary training would allow more opportunity to interact with healthcare providers from other disciplines, and for providers of those disciplines to work with chiropractic providers, potentially sowing seeds for interprofessional *teamwork* after graduation [24, 41–43].

Through the lens of a qualitative study designed to inform an upcoming chiropractic integration process, these results extend what is known about the perceived value and role of chiropractors within medical settings. While the lay public and chiropractic patients report fairly favorable assessments of chiropractic [5, 7, 10, 44], clinician attitudes often pitch toward more neutral or even negative views [3, 19, 22, 23]. The current study found generally positive support for the addition of a chiropractor to a specific clinical setting. This support came from patients, who anticipated that a chiropractor might help with their musculoskeletal pain, and providers, who hoped that the relief of this pain might allow patients to better focus on their other therapies. Though this organizational case study examined a unique, team-based, rehabilitation specialty hospital, the many qualities described by these participants are likely similar to those desired in chiropractors working in other multidisciplinary settings [3].

Among these many preferred qualities in a chiropractor, *patient-centeredness* was a central theme identified by all stakeholder groups in this study. Participants described patient-centeredness as rehabilitation care provided in a manner that was respectful, responsive to patient needs and values, personalized, evolving, and inclusive of the preferences of both the patient and their family. These characteristics are similar to narratives of integrated care patients who sought compassionate care that addressed their desires to be treated as a whole person with equal status as all other patients, and where patients were listened by an empathetic provider who offered continuity of care [45]. The extent to which patients view chiropractic care as patient-centered has had limited exploration. Older adults seeking integrated chiropractic and medical care reported a desire to be listened to, engaged in a doctor-patient relationship with good continuity of care, and receive treatment that enhanced patient safety [44]. Similarly, pregnant women emphasized the need for strong chiropractor-patient communication, along with a focus on safety [46]. Care coordination and patient-centered communication can be difficult to implement for patients with chronic pain [47, 48]. These challenges may be greater for patients with communication impairments from neurological injury. Future studies should continue to explore patient perceptions of chiropractic care in a variety of healthcare settings.

### Methodological rigor and study limitations

Our approach included multiple strategies designed to boost the methodological rigor of our qualitative organizational case study [49]. The credibility of our findings was enriched through prolonged engagement during site visits and persistent observation across work shifts, weekday/end schedules, and in all patient care units. Our four-person coding team allowed for continual peer debriefing as two or more members coded each transcript multiple times and the entire team engaged in discussion about the developing codebook. The completeness of our data benefited from the large number of persons interviewed for this project which allowed us to gather multiple perspectives from persons situated in varied roles throughout the organization. The dependability of our data analysis was enhanced through the use of a qualitative data management software which allowed audit trails of all coding decisions and queries to confirm the salience of our various themes across participant groups. Researcher reflexivity involved written annotations on personal experiences during data collection process and theoretical insights and coding decisions during analysis. Finally, the transferability of our findings is illustrated through the representative quotes offered in both the written text and the Additional file 1 which allows the reader to determine the applicability of these data to their own context. Additional file 2 provides information about the criteria for reporting qualitative research for this study.

Our study has limitations. While we used purposive sampling to elicit a broad range of stakeholder perspectives from a large number of persons engaged in this practice setting, our sample is not necessarily representative of all viewpoints. We only interviewed a small number of patients, as many had decreased abilities in expressive or receptive communication from their injuries. Family member perspectives were limited to those available during the site visit, which primarily included families who visited the facility on a daily basis. In some cases, the quality of the data gathered was limited by the circumstances inherent in conducting research in a clinical setting. For instance, interviews with nursing staff were curtailed in time and depth of investigation as patient care needs took precedent over the scheduled focus groups. Few facility administrators or board trustees were interviewed, in part due to timing of the site visit and in part due to access issues, which is not uncommon in qualitative studies [50]. Individuals from any of these stakeholder groups may have expressed different opinions regarding the preferred qualities in a doctor of chiropractic than those of the persons interviewed, which could limit the transferability of our findings. Further, these generally positive viewpoints might reflect participants’ efforts to offer socially desirable answers, particularly with investigators from a chiropractic research center who were newcomers to the rehabilitation hospital. We also did not collect or report descriptive statistics for demographic data or identify specific work roles from this relatively small healthcare facility to protect the privacy and confidentiality of participants and their interview responses.

## Conclusions

Our qualitative study provides a description of the professional and personal qualities preferred in a chiropractor by patients, families, clinical staff, and other stakeholders in an in-patient, rehabilitation setting. Study participants supported the addition of a chiropractor to the multidisciplinary team who practiced in a safe, evidence-based, patient-centered manner. Interprofessional skills that enhanced teamwork, intrapersonal qualities to support patients’ emotional journeys through the rehabilitation process, and an organizational perspective that amplified the mission of the institution also were desired. Rather than labeling stakeholder perceptions as good, bad or indifferent as in previous studies, these results highlight specific attributes chiropractors might cultivate to enhance patient outcomes and their experience of healthcare, influence clinical decision-making and interprofessional teamwork, and impact healthcare organizations. Chiropractic education might emphasize the development of such qualities in students who anticipate working in such collaborative care settings.

## Additional files

Additional file 1: Extended table of the qualities of the preferred chiropractor for multidisciplinary rehabilitation settings. (DOCX 33 kb)

Additional file 2: Consolidated criteria for reporting qualitative studies (COREQ) checklist. (DOCX 16 kb)

## Abbreviations

CMSH: Crotched Mountain Specialty Hospital
FG: Focus Group
Intvws: Interviews
LNA: Licensed Nursing Assistant
OT: Occupational Therapist
OTA: Occupational Therapy Assistant
PT: Physical Therapist
PTA: Physical Therapist Assistant
RN: Registered Nurse
ST: Speech Therapist
TR: Therapeutic Recreation Therapist
TRA: Therapeutic Recreation Assistants

## References

[1] Salsbury SA, Goertz CM, Twist EJ, Lisi AJ (2018). Integration of doctors of chiropractic into private sector health care facilities in the United States: a descriptive survey. J Manip Physiol Ther.

[2] Bronston LJ, Austin-McClellan LE, Lisi AJ, Donovan KC, Engle WW (2015). A survey of American chiropractic association members' experiences, attitudes, and perceptions of practice in integrated health care settings. J Chiropr Med.

[3] Lisi AJ, Khorsan R, Smith MM, Mittman BS (2014). Variations in the implementation and characteristics of chiropractic services in VA. Med Care.

[4] Christensen M, Hyland J, Goertz C, Kollasch M (2015). Practice Analysis of Chiropractic 2015: a project report, survey analysis, and summary of chiropractic practice in the United States.

[5] Weeks WB, Goertz CM, Meeker WC, Marchiori DM (2015). Public perceptions of doctors of chiropractic: results of a national survey and examination of variation according to respondents' likelihood to use chiropractic, experience with chiropractic, and chiropractic supply in local health care markets. J Manip Physiol Ther.

[6] Sanchez JE (1991). A look in the mirror: a critical and exploratory study of public perceptions of the chiropractic profession in New Jersey. J Manip Physiol Ther.

[7] Weeks WB, Goertz CM, Meeker WC, Marchiori DM (2016). Characteristics of US adults who have positive and negative perceptions of doctors of chiropractic and chiropractic care. J Manip Physiol Ther.

[8] Brown BT, Bonello R, Fernandez-Caamano R, Eaton S, Graham PL, Green H (2014). Consumer characteristics and perceptions of chiropractic and chiropractic services in Australia: results from a cross-sectional survey. J Manip Physiol Ther.

[9] Cherkin D, MacCornack FA, Berg AO (1989). Family physicians' views of chiropractors: hostile or hospitable?. Am J Public Health.

[10] Maiers M, Hondras MA, Salsbury SA, Bronfort G, Evans R (2016). What do patients value about spinal manipulation and home exercise for back-related leg pain? A qualitative study within a controlled clinical trial. Man Ther.

[11] Cambron JA, Cramer GD, Winterstein J (2007). Patient perceptions of chiropractic treatment for primary care disorders. J Manip Physiol Ther.

[12] Greene B, Smith M, Allareddy V, Haas M (2006). Referral patterns and attitudes of primary care physicians towards chiropractors. BMC Complement Altern Med.

[13] Mainous A, Gill J, Zoller J, Wolman M (2000). Fragmentation of patient care between chiropractors and family physicians. Arch Fam Med.

[14] Westin D, Tandberg T, John C, Axén I (2013). GPs opinions and perceptions of chiropractic in Sweden and Norway: a descriptive survey. Chiropr Man Therap.

[15] Allareddy V, Greene BR, Smith M, Haas M, Liao J (2007). Facilitators and barriers to improving interprofessional referral relationships between primary care physicians and chiropractors. J Ambul Care Manage.

[16] Greene B, Smith M, Haas M, Allareddy V (2007). How often are physicians and chiropractors provided with patient information when accepting referrals?. J Ambul Care Manage.

[17] Smith M, Greene B, Haas M, Allareddy V (2006). Intra-professional and inter-professional referral patterns of chiropractors. Chiropr Osteopat.

[18] Langworthy JM, Smink RD (2000). Chiropractic through the eyes of physiotherapists, manual therapists, and osteopaths in the Netherlands. J Altern Complement Med.

[19] Weis CA, Stuber K, Barrett J, Greco A, Kipershlak A, Glenn T, Desjardins R, Nash J, Busse J (2016). Attitudes toward chiropractic: a survey of Canadian obstetricians. J Evid Based Complementary Altern Med.

[20] Mullin L, Alcantara J, Barton D, Dever L (2011). Attitudes and views on chiropractic: a survey of United States midwives. Complement Ther Clin Pract.

[21] Wong J, Di Loreto L, Kara A, Yu K, Mattia A, Soave D, Weyman K, Kopansky-Giles D (2014). Assessing the change in attitudes, knowledge, and perspectives of medical students towards chiropractic after an educational intervention. J Chiropr Educ.

[22] Busse JW, Jacobs C, Ngo T, Rodine R, Torrance D, Jim J, Kulkarni AV, Petrisor B, Drew B, Bhandari M (2009). Attitudes toward chiropractic: a survey of north American orthopedic surgeons. Spine.

[23] Busse JW, Jim J, Jacobs C, Ngo T, Rodine R, Torrance D, Kulkarni AV, Petrisor B, Drew B, Bhandari M (2011). Attitudes towards chiropractic: an analysis of written comments from a survey of north american orthopaedic surgeons. Chiropr Man Therap.

[24] Salsbury SA, Goertz CM, Vining RD, Hondras MA, Andresen AA, Long CR, Lyons KJ, Killinger LZ, Wallace RB (2017). Interdisciplinary practice models for older adults with back pain: a qualitative evaluation. Gerontologist.

[25] GALLUP News. Americans rate healthcare providers high on honesty, ethics: GALLUP; 2016. http://news.gallup.com/poll/200057/americans-rate-healthcare-providers-high-honesty-ethics.aspx. Accessed 9 Feb 2018

[26] Cherkin DC, MacCornack FA (1989). Patient evaluations of low back pain care from family physicians and chiropractors. West J Med.

[27] Wong JJ, Di Loreto L, Kara A, Yu K, Mattia A, Soave D, Weyman K, Kopansky-Giles D (2013). Assessing the attitudes, knowledge and perspectives of medical students to chiropractic. J Can Chiropr Assoc.

[28] Vining RD, Gosselin DM, Thurmond J, Case K, Bruch FR (2017). Interdisciplinary rehabilitation for a patient with incomplete cervical spinal cord injury and multimorbidity: a case report. Medicine (Baltimore).

[29] Vining RD, Salsbury S, Cooley WC, Gosselin D, Corber L, Goertz CM (2018). Patients receiving chiropractic care in a neurorehabilitation hospital: a descriptive study. J Multidiscip Healthc.

[30] Stake RE (1995). The art of case study research.

[31] Crabtree BF, Miller WL (1999). Doing qualitative research.

[32] Lyons SS (2010). How do people make continence care happen? An analysis of organizational culture in two nursing homes. Gerontologist.

[33] Salsbury S, Vining R, Li Q, Thurmond J, Corber L, Gosselin D (2018). Social network analysis of a chiropractor during integration into the clinical staff of a rehabilitation hospital. J Chiropr Educ.

[34] Hsieh HF, Shannon SE (2005). Three approaches to qualitative content analysis. Qual Health Res.

[35] Boeije H (2002). A purposeful approach to the constant comparative method in the analysis of qualitative interviews. Qual Quant.

[36] Freshman B, Rubino L (2004). Emotional intelligence skills for maintaining social networks in healthcare organizations. Hosp Top.

[37] Walker BF (2016). The new chiropractic. Chiropr Man Therap.

[38] Nelson CF, Lawrence DJ, Triano JJ, Bronfort G, Perle SM, Metz RD, Hegetschweiler K, LaBrot T (2005). Chiropractic as spine care: a model for the profession. Chiropr Osteopat.

[39] Murphy D, Justice B, Paskowski I, Perle S, Schneider M (2011). The establishment of a primary spine care practitioner and its benefits to health care reform in the United States. Chiropr Man Therap.

[40] Goertz CM, Weeks WB, Justice B, Haldeman S (2017). A proposal to improve health-care value in spine care delivery: the primary spine practitioner. Spine J.

[41] Riva J, Lam J, Stanford E, Moore A, Endicott A, Krawchenko I (2010). Interprofessional education through shadowing experiences in multi-disciplinary clinical settings. Chiropr Osteopat.

[42] Seidman M, Vining R, Salsbury S (2015). Collaborative care for a patient with complex low back pain and long-term tobacco use: a case report. J Can Chiropr Assoc.

[43] Salsbury SA, Goertz CM, Twist EJ, Lisi AJ (2018). Integration of doctors of chiropractic into private sector health care facilities in the United States: a descriptive survey. J Manip Physiol Ther.

[44] Lyons K, Salsbury S, Hondras M, Jones M, Andresen A, Goertz C (2013). Perspectives of older adults on co-management of low back pain by doctors of chiropractic and family medicine physicians: a focus group study. BMC Complement Altern Med.

[45] Greenfield G, Ignatowicz AM, Belsi A, Pappas Y, Car J, Majeed A, Harris M (2014). Wake up, wake up! It's me! It's my life! Patient narratives on person-centeredness in the integrated care context: a qualitative study. BMC Health Serv Res.

[46] Sadr S, Pourkiani-Allah-Abad N, Stuber KJ (2012). The treatment experience of patients with low back pain during pregnancy and their chiropractors: a qualitative study. Chiropr Man Therap.

[47] Penney L, Ritenbaugh C, Elder C, Schneider J, Deyo R, DeBar L (2016). Primary care physicians, acupuncture and chiropractic clinicians, and chronic pain patients: a qualitative analysis of communication and care coordination patterns. BMC Complement Altern Med.

[48] Gulbrandsen P, Madsen H, Benth J, Laerum E (2010). Health care providers communicate less well with patients with chronic low back pain: a study of encounters at a back pain clinic in Denmark. Pain.

[49] Houghton C, Casey D, Shaw D, Murphy K (2013). Rigour in qualitative case-study research. Nurse Res.

[50] Harvey WS (2011). Strategies for conducting elite interviews. Qual Res.

