# Supplementary material for: Be good, communicate, and collaborate: a qualitative analysis of stakeholder perspectives on adding a chiropractor to the multidisciplinary rehabilitation team
Source: Chiropr Man Therap. 2018 Jun 22;26:29. doi: 10.1186/s12998-018-0200-4 (PMC6014012; doi:10.1186/s12998-018-0200-4)
Supplement: Supplementary file 1 — Extended table of the qualities of the preferred chiropractor for multidisciplinary rehabilitation settings. (DOCX 33 kb) [file 12998_2018_200_MOESM1_ESM.docx]

| **Domain**/Theme | **Definition/**Representative Quotes (Participant Identifier) |
| --- | --- |
| **Patient-Centeredness** (Central Domain) | **Characteristics of the chiropractor (and all staff members) that demonstrate the provision of care that is respectful and responsive to the patient’s values, preferences and needs.** |
|  | Patient oriented. He [therapist] makes you part of the program. You know exactly what’s going on and why he is doing what he’s doing. (P1 – Patient)  You would be talked to about your condition a little bit more and what their [staff] plan is to do for you. (P2 – Patient)  They’d [the chiropractor] have to know their [the patient] limitations and their desire to maybe be limbered up a little bit with exercise and different movements. (P3 – Patient)  Just to get along with them [the patients]. Don’t argue with them. (P4 – Patient)  Every patient here has their own story, so what is good for one person may not be good for another person. (P5 – Patient)  The cause of them being here. How long they expect to be here. I wouldn’t want a doctor or a chiropractor to come and only have the patient for a week and then they’d be gone. I think a chiropractor needs more time with a patient than just a short amount of time. (P6 – Patient)  Someone that he [my father] feels comfortable with that he could… build a relationship with, I guess, is the best way to put it, and get familiar with…and the hands-on and building a relationship with the patients. (FA – Family Member)  When you’re dealing with folks with brain injuries, it’s really important to not only ask the patient what might be needed or how the approach might be, but I think it’s really good to check in with the family members, too, to see if that would be a good thing, especially for brain injury. Sometimes things have got lost in the translation. (FC – Family Member)  Patients here, you get the people that are very satisfied with the care and then you get the opposite, that are very dissatisfied with the care. People that are dissatisfied are generally so because they don't feel like they're getting what they need. (M2 – Medical Staff)  You have to be willing to put your own agenda behind what needs to happen for that patient on that given day. (M6 – Medical Staff)  You gain a little bit of perspective of the patient situation and maybe you look at it from a totally different direction, and suddenly that frustrating behavior, frustrating situation, you have a different answer for. You can approach it from a different perspective and maybe solve the problem so that the frustration doesn’t exist. (NL4 – Nursing Leaders)  Trying to find someone who will fit in here and really keep the needs of patients in the fore. (NL4 – Nursing Leaders)  Different times for different people, you know, some people are morning people, some people are later in the day people. (NMU1 – Nursing Staff)  One thing we forget a lot of times doing their care, it is not our pace, it is their pace. People coming from the outside in, it is one of the hardest things to learn. It is not about us, it is about them [the patients]. That person can do it in two minutes. That one takes ten. You need to give them that, because you’re knocking them down a peg when you don’t. Everyone is different, there’s no two cases alike here, no two alike. (NMU1 – Nursing Staff)  There is variability not just patient to patient but within the same patient as they may not be consistent. (T7 – Therapy Staff)  It will vary from patient to patient. Like you mentioned with narcotic use…communication disorder, the ability to report accurately and give feedback that you’re looking for will be a challenge for each patient. (T1 – Therapy Staff) |
| **Professional Qualities** | **Characteristics of the chiropractor that demonstrate his or her clinical knowledge and competence and proficiency in the specialized field.** |
| - Clinical Acumen | To understand what my specific therapeutic requirements are over and above what I am able to currently receive here. (P1 – Patient)  They’d [chiropractor] have to know their [patient] limitations and their desire to maybe be limbered up a little bit with exercise and different movements. (P3 – Patient)  To take care of pain issues (P4 – Patient)  You need experience with brain injury…The way cognition and communication are affected are going to be a big factor, especially after looking for feedback on how the adjustment went. (T8 – Therapy Staff)  If they [chiropractors] could control pain and if they know how to manage tone and they work with that but I don’t know that chiropractors have that neuro-muscular piece. (T2 – Therapy Leaders)  Thinking about how to be able to properly position someone, how to get them so that you’re able to do an adjustment. To sometimes even be able to get into their personal space to do an adjustment. Some of our guys are very big, they can’t move. To try to transfer them. (T1 – Therapy Leaders)  Know when to give the adjustment and when not to. Sometimes the chiropractor has to leave it alone… He or she should have extreme confidence in the ability to give a chiropractic adjustment correctly. (C1 – Community Member)  I think the doctor would know if they can treat or not treat. I think depends on what the doctor decides that they're comfortable doing adjustments for the patient. But that's more of a clinical question than a concern that I would have for the patient. (A2 – Administrative Staff)  A lot of patients with head injuries have a difficult time controlling their behaviors and their moods and emotions and I think that would be a challenge potentially…with the hands-on care that chiropractic care involves…there are just so many different presentations here, you'd have to really pick the people that are suited to get that modality. I don't think everybody would be a candidate. (M2 – Medical Staff) |
| - Efficacious Treatment | The first starting point is that it [chiropractic] will have immediate value, or direct value, in comfort, capacity, and functional opportunity for individuals. (A1 - Administrative Staff)  Something as simple as my chiropractor adjusts my wrists…my dad complained about pain in his wrists when he’s pulling on the parallel bars. I could see that maybe benefitting my dad. Doing an adjustment… if it’s something that is going to help you with your wrists and your pain. (FA - Family Member)  There’s lot of people here with pain. You got the spinal cord injuries, they don’t get to move around at all… Think about sitting in a chair all the time, you get all twisted up. I think it would be great to have somebody to be able to help these patients, definitely. (FD - Family Member)  They [patients] get better…do that thing he [chiropractor] does. Hopefully he can put them back in place and make them work better… (P4 – Patient)  I guess lots of tools in your repertoire of addressing one problem. (T2 – Therapy Staff)  It'll be nice to treat a lot of the pain stuff or the musculoskeletal stuff with other modalities… People are excited about things that can help them. (M2 - Medical Staff)  The successful integration of a chiropractor would be that they had something to offer to the patient that would help them on that pathway. (M5 – Medical Staff) |
| - Safe Practitioner | So that would be the thing I’d be afraid of, is getting the chiropractor even to come close to some people without it hurting more. (P2 – Patient)  Just to know that the chiropractor…would have enough knowledge of the extent of their [patient] injury and their physiology that the chiropractor would work with a patient to improve…I don’t want to use the word hurt, but just work with them in a way that would be least invasive…and, you know, would benefit… (FA – Family Member)  Well, my only concern would be is this, would you use the snap, crackle? I don’t know about that one. (C2 – Community Member)  My concern would be that especially where you have patients with neurological issues, spinal issues, whatever their problem happens to be, that the assessment be thorough and adequate and discussed and decided upon in conjunction with medicine to make sure that the patient still is kept safe. (NL2 – Nursing Leaders)  We have a lot of spinal cord injuries here so that crosses your mind. Can anything happen to their spine during the process or being adjusted? I think it kind of outweighs the benefits, I think it would be beneficial here as long as it would be safe for a spinal cord injury patient. (NMU5 – Nursing Staff)  The nerve-wracking part of it, because we have a lot of people that have spinal cord swelling. I feel like if, I don't know, just one wrong movement could cause more damage instead of relief. (NH3 – Nursing Staff)  An adjustment, even with an instrument, can be a little bit abrupt sometimes and positioning might be uncomfortable and they [patients] might not understand the point. So I guess the concern is more not the chiropractic care or the manipulation being the problem, but integrating it appropriately to the right people at the right time, and understanding that sometimes there's going to be an adjustment that…might hurt them or you might increase some tone for a minute. (M1 – Medical Staff)  Being safe with manipulations on people who’ve had spinal cord injuries. (M3 – Medical Staff)  My biggest concern is making sure whoever's touching these folks understand they have underlying anatomy and neuro as well as all of us can, so that we do no harm. (M6 – Medical Staff) |
| **Interpersonal Qualities** | **Characteristics of the chiropractor that will enhance his or her ability to work with neurorehabilitation patients and their families** |
| - Comforting Patience | [My son] needs to feel comfortable, he needs to feel like he can trust that person…They’re going to have to be caring and loving and take time with the patients. I think they’ll do really well. As long as they [the patients] trust the person and it takes a little while. I think they’re going to have to do it a little... Not come in the first day and start working really hard on them. (FD – Family Member)  I think that maybe starting by something even as simple as adjusting his wrists might help him. Slowly and approaching it very gingerly, yes… having a dedicated office or area where they could take the patients to that would be calming…the one thing that would definitely be beneficial is a very calm, quiet area where the patients could have their session with the chiropractor. (FA – Family Member)  They’d [the chiropractor] better have some good patience, that’s all I can say, is they’re going to have to have patience with it. (P2 – Patient)  Being here and having somebody value my knowledge and my presence. (M1 – Medical Staff)  Stimulation, being able to not be over stimulating and knowing when to speak and when not to because it is very important. (T3 – Therapy Staff) |
| - Familiar Connections | Knowing the staff and the patients and building a rapport with them and respect…an open, honest relationship… getting familiar with the patient through talking to the staff members before working with them. (FA – Family Member)  I would like to see him [my son] have more…familiarity, so he gets comfortable with somebody. (FD – Family Member)  I would say behavior because the pain, we deal with it here every day. So you don’t really think about it. But for somebody to walk in, they would try to do what we would do and it would be totally disastrous because we built a rapport with the person. (NMU1 – Nursing Staff) |
| - Emotional Intelligence | Have a thick skin, don’t take anything personally…don’t take yourself too seriously for one, and don’t take the patient too seriously…the patient will have this anger, it’s being directed at you, but it’s not about you…remember this isn’t about them calling you whatever they’re calling you, it’s not them yelling at you for the day, it’s the fact that they can’t help what they do because of their brain injury…you have to remember that when you work with this population. (NL5 – Nursing Leader)  We deal with a lot of stroke victims who have one side weak and…they are still very coherent…really understand the frustration…so there’s a lot of resentment. Not necessarily towards staff, it is totally about their circumstances knowing there is nothing they can do or feeling there is nothing they can do and that is their reality for the rest of their life. I can see that as being a huge hurdle especially if you bring in a chiropractor who doesn’t deal with behaviors. (NML3 – Nursing Staff)  You don’t want to go in with a pre-conceived notion…oh [I’ve] been doing this for a year, [I] know… what the story is. Because sometimes they’re stuck and they’re very sensitive, very, very emotional. And they can be verbally aggressive. (TL1 – Therapy Leader)  It’s a population that’s very difficult because of the level of difficulty and the slow rate of progress. It’s not a population that gives a therapist that direct reward for so much better after six weeks of you. (TL1 – Therapy Leaders)  These folks show up angry and despairing with strong patterns in their life of self-destructive or victim experiences. For practitioners who aren’t familiar with, and not just accepting, but even embracing, of individuals of those histories and ways of speaking, ways of relating, it can be unpleasant work. But for those of us with more experience with it, it can be really kind of wonderful work, and very fulfilling, very meaningful, very purposeful work. (A1 - Administrative Staff)  We think about people's personalities and…what they're likely to do and not do and likely to agree with or not agree with, what kind of people they are…that's valuable for anybody treating, working with somebody. It's a human, intuitive, skill. If you're in the healthcare field you probably have that skill on some level. (M2 – Medical Staff)  Our patients' days…they're already working so hard to make sense of what is occurring that if somebody comes in and manhandles a situation, it can cause distrust that's deep-seated for a while. (M6 – Medical Staff) |
| **Interprofessional Qualities** | **Characteristics of the chiropractor that will enhance his or her ability to work with other healthcare professionals within an interdisciplinary setting** |
| - Teamwork | Just to work well together, become part of a synergistic program. (P1 – Patient)  Be good and be able to communicate with a team and do their job well and collaborate. I think that would go a long way. (M2 – Medical Staff)  The primary goal would be having an interdisciplinary approach to care, to make sure that everybody that has to be involved is involved, and knows what everybody else is doing, so that everything’s working together to head the patient in the direction they need to go. (NL2 – Nursing Leader)  We’re 95% team oriented here. We do everything as a team, even across shifts, so it’s all about team play here. (NML3 – Nursing Staff)  A concern I have on a daily basis is just that because our teams are so large and there are so many different people coming at these problems from different places, it’s always important that everybody be informed. (TL1 – Therapy Leader)  We have an incredible crew, we have people that do this on a daily basis and that is key for the resident because if something is blocking their recovery that’s one way you fix it. (NMU2 – Nursing Staff)  Someone who is willing to be a part of a team because I think we have a pretty great one so being able to join in the team and wanting to share their thoughts and feelings towards that. (T3 – Therapy Staff)  As chiropractors, are they comfortable co-treating, like with physical therapy or occupational therapy or speech because there are a lot of times when it does need two of us. (T1 – Therapy Staff)  The whole effort here, when chiropractic goes on here, there should be a unity of integrated health services and…everybody gets credited with the results. (C1 – Community Member)  They’ve [the chiropractor] got to realize that they’re going to have to really team with the therapists, because they’re in a way, sort of the competition, they are the other hands-on treatment. So that they’re not taking over. (M3 – Medical Staff) |
| - Resourcefulness | Sometimes you go in with a very good game plan and you have to completely change so you have to be very flexible to work here… If you’re really going in with a plan, that’s not good, and you can’t mentally flex that plan in your head that’s not going to be a good fit. (T4 – Therapy Staff)  It’s a very diverse population. Nobody’s brain heals exactly the same from an injury so you’re constantly challenged by the presentation, so clinically, it’s very interesting. (T1 – Therapy Staff)  A lot of different modalities are tried. People aren’t really geared here to give up. I think that’s the biggest thing, they’re not geared to give up, nobody ever gives up. (NL1 – Nursing Leader)  I think maybe just an openness to seeing things differently and not just sticking to the protocol, within reason, and obviously, within the bounds of safety for the patient first. (M1 – Medical Staff)  You're going to see somebody and then you're going to figure out how their disability is getting in their way. (M6 – Medical Staff)  He better be able to drive up the mountain road in snow. (NL3 – Nursing Leader) |
| - Openness to Feedback | A willingness to ask questions and get feedback. (T7 – Therapy Staff)  We have continuous give and take feedback between all the different groups of like staff, the therapists, the doctors, the nurses so if that person is suffering pain during their therapy or after therapy or at any time during the day there is always a doctor and you can let them know. They [clinical staff] have rounds twice a week so they can get updated on how the person’s main regimen is affecting them. (NH2 – Nursing Staff)  An openness to learn because you’re going to learn so much more…you’ve got to be open because you may have never done anything like that in the past, doesn’t mean it doesn’t work that way and it might work quite well here. (TL2 – Therapy Leader)  It would be my expectation that that person [chiropractor] would come to the same meetings that we all go to so that they can ask questions, learn our patients, know who does what and would be another addition to the team as opposed to working out there by themselves, and those meetings are what give you all of the feedback from all the players, so from nursing to the physician to the housekeeping concern to behaviors, all of it is at those meetings. (M5 – Medical Staff) |
| **Organizational Qualities** | **Characteristics of the chiropractor and all staff members that are considered important attributes for working in this particular healthcare organization** |
| - Personality Fit | There are a lot of opinions on this mountain, and there are a lot of other strong personalities on this mountain. (NL1 – Nursing Leader)  What’s not happening is opening this up so that any chiropractor can come up here and adjust any patient. What’s happening is we’re trying to find someone who is going to fit with this team. There’s going to be a variety of people involved in figuring out who that person is. (NL1 – Nursing Leader)  They better have a sense of humor because that is what keeps us going is a pretty solid sense of humor. (NML3 – Nursing Staff)  He’s got to have a personality. (T2 – Therapy Staff)  Finding joy in the smaller pieces, smaller rewards, is not something that everybody can do. (TL2 – Therapy Leader)  Well I think it'll have to be an unselfish person. (C1 – Community Member)  Personality is key. It’s going to need to be somebody who is going to interact well with the staff, will listen to the staff’s concerns, and will feel comfortable educating the staff because none of us know anything about chiropractic, so that we can slowly develop a comfort level with it. (M5 – Medical Staff) |
| - Institutional Compliance | Staff has to have the chiropractor aware of what they can and can’t do when they start investigating somebody’s health insurance. If the insurance says, you can’t work on that side of the body or you can’t work on that part of the body, then you can’t. It won’t be covered by insurance. (P2 – Patient)  Significant use of technology, some of that being rehab technology, some of it being…electronic medical records, etc. High emphasis on quality improvement systems as the…decision-supported, decision-driver that we use. (A1 –Administrative Staff)  To say, “Well, what does a good chiropractor do?” I would not be able to look at a chiropractic chart and say, “this is basic standard of care or this falls out”…I wouldn’t know exactly. (M3 – Medical Staff)  When we were looking at how to privilege chiropractors coming here, we looked at a couple of hospitals…One thing that we took out because that was not perceived well was the chiropractor being able to order physical therapists to do things. You know, some of our physical therapists are PhD level here. (M3 – Medical Staff)  It would be important for this person to come to the various groups that are already set up, for example, we do psych rounds on Tuesdays, we do physiatry rounds on Thursdays, we do the team meetings every day at 11, interdisciplinary team is always Thursday at 8:30, because these are the systems that we’ve put in place to make sure that communication is open, all of those issues are discussed at those meetings. (M6 – Medical Staff) |
| - Mission Alignment | Once you get past all of that, there’s on driving force on the mountain, and that is to make a positive impact on this person’s life as they try to recover from something that is very hard and a long journey. And we know the journey doesn’t end here. They [the chiropractor] can’t think that they can fix something, fixing something is not what we do. (NL1 – Nursing Leader)  I find that my personal mission as a clinician and [the facility’s] mission to serve the underserved, to serve…it’s related to the mission and the population. It’s always the most difficult [patients] it’s seeing [the institution] answer the call of the community as it has changed through the years. (TL2 – Therapy Leaders)  The people that work there. I don’t care if it’s from the president and down to someone that’s sweeping a floor, they just are wonderful, wonderful people. You can just feel it when you walk in there. It’s just such a relaxed atmosphere. The good they do, it’s just remarkable. It totally is. They take people in that nobody else wants to work with or try to help. That’s what we like about it. It’s just doing such good work for so many people. (C2 – Community Member)  [Our mission] is a lifelong alliance is the way we refer to it. It’s approached with a high degree of commitment to indigent individuals who often have very difficult lives and life changing events. (A1 – Administrative Staff) |
