# Supplementary material for: Be good, communicate, and collaborate: a qualitative analysis of stakeholder perspectives on adding a chiropractor to the multidisciplinary rehabilitation team
Source: Chiropr Man Therap. 2018 Jun 22;26:29. doi: 10.1186/s12998-018-0200-4 (PMC6014012; doi:10.1186/s12998-018-0200-4)
Supplement: Supplementary file 2 — Consolidated criteria for reporting qualitative studies (COREQ) checklist. (DOCX 16 kb) [file 12998_2018_200_MOESM2_ESM.docx]

Supplementary Materials - Consolidated Criteria for Reporting Qualitative Studies (COREQ) Checklist

| **Domain 1: Research Team and Reflexivity** | | |
| --- | --- | --- |
| **Personal Characteristics** | | |
| Interviewer/ Facilitator | Who conducted interviews? | Stacie A. Salsbury, PhD, RN  Robert D. Vining, DC |
| Credentials | What were the researchers’ credentials? | PhD-prepared qualitative researcher with experience in organizational culture (Salsbury)  Doctor of chiropractic with extensive experience in clinical practice and clinical research (Vining)  Doctor of chiropractic and PhD-prepared health services researcher (Goertz)  Physical therapist with long-term experience at research setting (Gosselin) |
| Occupation | What were the researchers’ occupation(s) at time of study? | Clinical research faculty at Palmer Center for Chiropractic Research (Salsbury, Vining)  Vice Chancellor of Research and Health Policy at Palmer College of Chiropractic (Goertz)  Physical therapist at Crotched Mountain Specialty Hospital (Gosselin) |
| Gender | Researcher genders? | Research team included members who identified as female and male genders. |
| Experience/ Training | Experience and training of researchers? | As described above |
| **Relationship with Participants** | | |
| Relationship Established | Researcher established relationship with participants before start of study? | At this first series, the interviewers had no-to-minimal previous contact with most participants before the start of study. One researcher (Gosselin) provided clinical care for patients and was a colleague of organizational stakeholders, but was not involved in conducting the interviews or the primary analysis. |
| Participant Knowledge of Interviewer | What did participants know about researchers? | Introduction by institutional representatives, details in informed consent, and description of role and training by researchers before start of interview. |
| Interviewer Characteristics | What researcher characteristics reported to participants? | Role, training, professional credentials, workplace, experience in clinical research. |
| **Domain 2: Study Design** | | |
| **Theoretical Framework** | | |
| Methodological Orientation & Theory | Methodological orientation underpins study? | Organizational case study approach using ethnographic methods |
| **Participant Selection** | | |
| Sampling | How were participants selected? | Purposive sampling |
| Method of Approach | How were participants approached? | Face-to-face contact by institutional representatives, brochures |
| Sample Size | How many participants were in the study? | Sixty participants, including 48 staff members, 6 patients, 4 family members, and 2 community members. |
| Non-participation | How many people refused to participate or dropped out? Reasons? | Refusals to participate were not collected systematically. Staff non-participation reasons included patient care delivery at time of interview. No drop outs. |
| **Setting** | | |
| Setting of Data Collection | Where was the data collected? | Hospital workplace in conference rooms, offices, patient rooms or unoccupied lounges. |
| Presence of Non-participants | Was anyone else present besides the participants and researchers? | Non-participants were not present at time of interview. |
| Sample Description | What are the important characteristics of the sample? | Demographics were not collected on individuals; setting role (patient, staff, family) reported. |
| **Data Collection** | | |
| Interview Guide | Were questions, prompts, guides provided by the authors? Was it pilot tested? | A semi-structured interview manual guided the sessions, with interview topics varying by participant role. Reviewed by both research staff and clinical partners, but not pilot-tested with similar participants before use in setting. |
| Repeat Interviews | Were repeat interviews carried out? If yes, how many? | Many clinical/administrative staff and a few patients were interviewed up to 3 times over a 2-year time period. Most patient and family interviews were single occurrence due to patient discharge. This study reports only on baseline interviews. |
| Audio/visual Recording | Did the research use audio or visual recording to collect the data? | Audiorecorded with digital recorders. |
| Field notes | Were field notes made during and/or after the interview or focus group? | Written fieldnotes documented participants’ seat position, gender and role during group interviews to aid with transcription. Brief, oral fieldnotes were recorded post-interview to clarify points or comment on theoretical/methodological points. |
| Duration | What was the duration of the interviews or focus group? | Focus group interviews lasted up to 1 hour. Individual interviews with family and staff lasted between 20-40 minutes, with some staff interviews lasting 1 hour. Patient interviews were shorter in duration (15 minutes) to decrease participant burden. |
| Data Saturation | Was data saturation discussed? | Data saturation not discussed. Credibility and completeness of findings noted in Discussion section. |
| Transcripts Returned | Were transcripts returned to participants for comment and/or correction? | Transcripts were not returned to participants for comment and/or correction. |
| **Domain 3: Data Analysis and Findings** | | |
| **Data Analysis** | | |
| Number of Data Coders | How many data coders coded the data? | 4 coders coded the data. |
| Description of Coding Tree | Did authors provide a description of the coding tree? | Superordinate (parent nodes or domains) and subordinate (child nodes or themes) categories were developed as links between the various codes were identified. |
| Derivation of Themes | Were themes identified in advance or derived from the data? | Themes were identified inductively from the data during analysis. |
| Software | What software, if applicable, was used to manage the data? | NVivo® (Version 9.2, QSR International Pty Ltd, Victoria, Australia) |
| Participant Checking | Did participants provide feedback on the findings? | Participants did not provide feedback on the findings. |
| **Reporting** | | |
| Quotations Presented | Were participant quotations presented to illustrate the themes / findings? Was each quotation identified? | Quotations to illustrate themes presented by participant role and transcript ID in narrative text and supplementary materials. |
| Data/Findings Consistent | Was there consistency between the data presented and the findings? | Yes. In addition, supplementary materials provide evidence of consistency between at and reported findings. |
| Clarity Major Themes | Were major themes clearly presented in the findings? | Yes, in written narrative, table, and figure. |
| Clarity Minor Themes | Is there a description of diverse cases or discussion of minor themes? | Yes, in written narrative, table, and figure. |

<https://academic.oup.com/intqhc/article/19/6/349/1791966/Consolidated-criteria-for-reporting-qualitative>
